# Mimotopes selected with a neutralizing antibody against urease B from *Helicobacter pylori *induce enzyme inhibitory antibodies in mice upon vaccination

**DOI:** 10.1186/1472-6750-10-84

**Published:** 2010-11-30

**Authors:** Yan Li, Yunshan Ning, Yundan Wang, Dandan Peng, Yaodong Jiang, Lili Zhang, Min Long, Jun Luo, Ming Li

**Affiliations:** 1 School of Biotechnology, Southern Medical University, Guangzhou Dadaobei No.1838, Guangzhou, China, 510515; 2 Department of Cardiology, Guangzhou General Hospital of Guangzhou Military Command, Guangzhou, China; 3 Department of Urology, Nanfang Hospital, Southern Medical University, Guangzhou, China; 4 Cancer Center, Nanfang Hospital, Southern Medical University, Guangzhou, China; 5 Department of Microbiology, Southern Medical University, Guangzhou, China

## Abstract

**Background:**

Urease B is an important virulence factor that is required for *Helicobacter pylori *to colonise the gastric mucosa. Mouse monoclonal antibodies (mAbs) that inhibit urease B enzymatic activity will be useful as vaccines for the prevention and treatment of *H. pylori *infection. Here, we produced murine mAbs against urease B that neutralize the enzyme's activity. We mapped their epitopes by phage display libraries and investigated the immunogenicity of the selected mimotopes *in vivo*.

**Results:**

The urease B gene was obtained (GenBank accession No. DQ141576) and the recombinant pGEX-4T-1/UreaseB protein was expressed in *Escherichia coli *as a 92-kDa recombinant fusion protein with glutathione-S-transferase (GST). Five mAbs U001-U005 were produced by a hybridoma-based technique with urease B-GST as an immunogen. Only U001 could inhibit urease B enzymatic activity. Immunoscreening via phage display libraries revealed two different mimotopes of urease B protein; EXXXHDM from ph.D.12-library and EXXXHSM from ph.D.C7C that matched the urease B proteins at 347-353 aa. The antiserum induced by selected phage clones clearly recognised the urease B protein and inhibited its enzymatic activity, which indicated that the phagotope-induced immune responses were antigen specific.

**Conclusions:**

The present work demonstrated that phage-displayed mimotopes were accessible to the mouse immune system and triggered a humoral response. The urease B mimotope could provide a novel and promising approach for the development of a vaccine for the diagnosis and treatment of *H. pylori *infection.

## Background

*Helicobacter pylori *is a helical Gram-negative bacillus that was originally discovered by Marshall and Warren in the stomach of patients with gastritis and peptic ulceration [1]. *H. pylori *has subsequently been recognised as the major aetiological determinant of various gastroduodenal diseases. Approximately half of the world's population has been estimated to be infected by *H. pylori *and harbours the bacterium in their upper gastrointestinal tract [2]. Even though antibiotic-based triple therapy is still the most effective treatment for *H. pylori *infection, it seems that it is not feasible for large-scale control of infection, partly because of the high cost, poor compliance, and emergence of antibiotic-resistant strains. Increasing rates of therapeutic treatment failure and high rates of re-infection, together with low hygiene standards in developing countries have made it imperative to develop vaccines to control infection [3].

Currently, most *H. pylori *vaccines in animal models have utilised whole-cell preparation of native or recombinant proteins from the bacterium, together with mucosal adjuvant. In general, these vaccines are designed from a natural form of the pathogen after lysis or inactivation that differs from natural epitopes [4]. In response to *H. pylori *infection, the host triggers vigorous humoral and cellular immune responses. Although *H. pylori-*specific antibodies have been detected at high titres in inflamed gastric mucosa and in the serum, the infection can persist and/or never resolve. This suggests that *H. pylori *can evade the innate and adaptive immune responses, and the latter responses triggered by *H. pylori *via this natural approach do not elicit effective immunity [5]. Therefore, we hypothesise that modified immunity might be achieved via the use of mimotopes that differ from natural epitopes. This approach might be able to trigger an effective immune response that is absent in natural infections and natural-immunity-based approaches.

Phage display peptide libraries are usually employed to select epitopes, which mimic the epitopes of natural proteins recognised by the immune system. Such mimotopes are widely used in the development of vaccines against many diseases [6-8], the design of molecules that act as agonists or antagonists to many key biomolecules, and the development of diagnostic reagents [9-12]. It has been reported that mimotopes induce production of protective antibodies, and consequently, become candidates for the development of potential vaccines [13,14]. Mimotopes selected from random peptide libraries can drive an active immune response towards the original antigen and lead to effective immunity [15-17].

Urease plays a central role in the pathogenesis of *H. pylori *infection and promotes colonisation of the stomach and gut. Urease enzymatically hydrolyses urea to form ammonia and bicarbonate, which neutralise gastrointestinal acids and protect the bacteria against the acidic environment of the stomach. Urease is composed of two major subunits, urease A and urease B, and the latter is considered to be an excellent antigen for the induction of protective immune responses [18,19]. Mucosal vaccination with *Lactococcus lactis *that expresses urease B induces the production of IgG in blood and urease-B-specific faecal IgA against *H. pylori *infection [20]. Recently, by transformation of the gene of urease B into carrot, Zhang *et al. *have found that transgenic carrot plants can express the protein of urease B and effectively induce immune responses in mice [21]. In addition, immunisation of mice with the trivalent fusion vaccine that was constructed by genetically linking heat shock protein A (HspA), *H. pylori *adhesion (HpaA) and urease B414 (250-387 aa), has been shown to protect mice from *H. pylori *infection [22]. Thus, urease B appears to be an important target for the design of a prophylactic vaccine for *H. pylori*.

Using this technique, we have already obtained the neutralising mimotopes of Lpp20 and catalase for *H. pylori *[23,24]. Whether such mimotopes of urease B can be exploited to elicit functional antibody responses against *H. pylori *has yet to be fully investigated.

In the present study, we applied the phage display technology to identify the neutralising epitope of *H. pylori *urease B with specific monoclonal antibody (mAb) U001 that has been shown to inhibit significantly urease activity. Two phage-displayed libraries were immunoscreened and the selected phage clones were analysed. The mimotopes were used to immunise BALB/c mice and the immune responses for the phagotopes were evaluated.

## Results

### Expression and purification of recombinant protein

**Figure 1:**
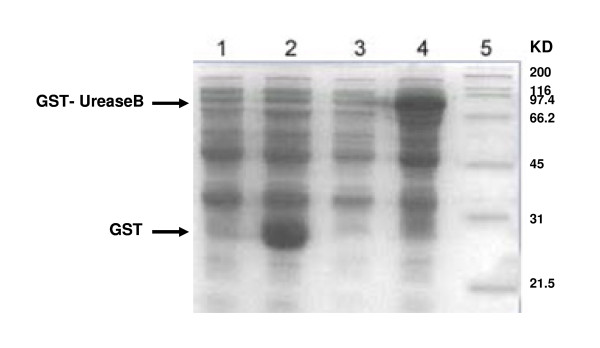
**SDS-PAGE analysis of expression of pGEX-4T-1/urease B in *E. coli***. 1: pGEX-4T-1 before induction; 2: pGEX-4T-1 after induction; 3: pGEX-4T-1/urease B before induction; 4: pGEX-4T-1/urease B after induction; 5: protein marker.

**Figure 2:**
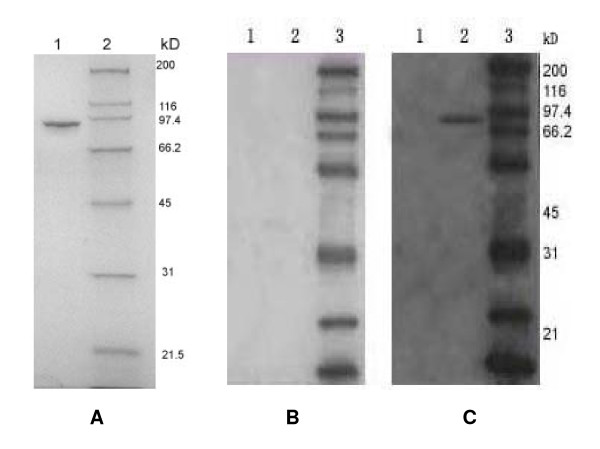
**SDS-PAGE analysis of purified urease B and Western blot analysis of urease B reactivity with patient serum**. (A) Bacteria expressing GST-urease B were lysed by sonication, and the supernatant was applied to a Glutathione Sepharose 4B column. Purified urease B was analysed on a 10% SDS-PAGE. 1: purified urease B; 2: protein marker. (B and C) The gels were transferred to nitrocellulose membranes and probed with diluted human sera (1:50) from a normal donor or an *H. pylori*-infected patient. Detection was performed with a goat anti-human antibody linked to HRP (1:10,000), followed by staining with 3-amino-9-ethylcarbozole. 1: GST; 2: urease B; 3: protein marker.

Genomic DNA extracted from *H. pylori *was used as a template for PCR. To amplify the DNA that encoded urease B, which included a major part of the active site, primers were designed as described in the Methods. An aliquot of the PCR mixture was analysed by agarose gel electrophoresis and a DNA fragment of about 1700 bp was detected, which corresponded to the expected position of urease B. The DNA sequence was submitted to GenBank (accession No. DQ141576). Expression of recombinant protein urease B-GST was harvested from *Escherichia coli *(Top10) and analysed by SDS-PAGE. We observed a band of 92 kDa, which corresponded to the predicted molecular size of the fusion protein (Figure 1). Soluble urease B-GST protein was purified by glutathione Sepharose (Figure 2) and used as the immunogen. Western blotting confirmed that sera from an *H. pylori*-infected patient specifically recognised the recombinant urease B protein, whereas the negative sera could not (Figure 2).

### Production and characterisation of anti-urease B mAbs

Five hybridomas, U001-U005, were obtained by cell fusion. All mAbs were IgG1 (κ) and showed high specificity to urease B. The titre of culture supernatant and ascites fluid was 1:32-1:64 and 1:32,000-1:128,000, respectively. Triple serial concentrations of the antigen and each mAb were selected to construct the corresponding curves and to extrapolate the K_aff _values using the formula given in the Methods section. The apparent K_a _values for the five mAbs varied from 1×10^8^/M to 1×10^12^/M. mAb U001 displayed the highest affinity of ~10^12^/M (data not shown).

Inhibition and competition studies using increasing concentrations of inhibitor or competitor mAbs revealed that four of the five mAbs (U001, U003, U004 and U005) competed significantly with each other, which indicated recognition of the same or overlapping epitopes. However, U002 did not compete with the other antibodies and recognised a distinct epitope.

**Figure 3:**
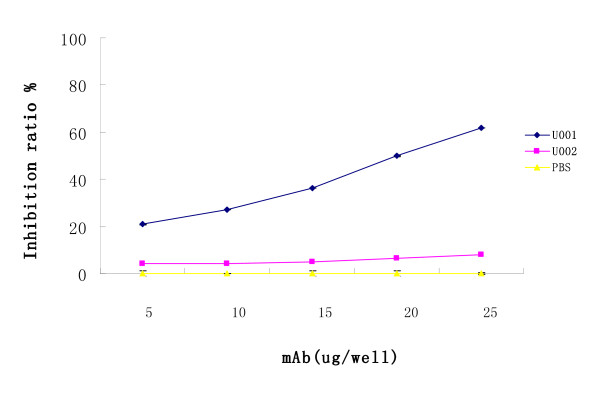
**Inhibition of urease B enzymatic activity by specific mAb U001**. Urease (25 μl) was incubated with 25 μl mAb U001 or U002 (equivalent to 0-25 μg) in 96-well microtitre plates overnight at 4°C, with PBS as a control. On the following day, 50 μl 50 mM phosphate buffer (pH 6.8) that contained 500 mM urea, 0.02% phenol red, and 0.1 mM DTT was added to each well. The colour development was monitored at 550 nm with a microplate reader. The inhibition ratio was determined by the following equation: [(activity without Ab - activity with Ab)/(activity without Ab)] × 100.

Urease B enzymatic activity was measured and the inhibitory effects of U001 and U002 were quantitated by means of an inhibition assay. The inhibition rates were calculated by comparing mAb-treated and control rates for Urease B activity (Figure 3). U001 possessed the strongest inhibitory effect on urease B enzymatic activity, whereas mAb U002 had a very moderate effect.

### Screening of phage display peptide libraries and characterisation of recombinant phage

**Figure 4:**
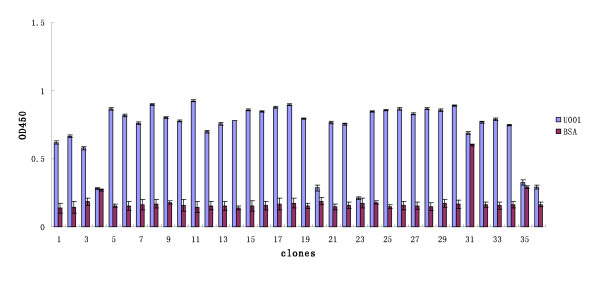
**Binding specificity of selected phage from the Ph.D.-12 library**. Wells of 96-well microtitre plates were coated with mAb U001 and BSA (10 μg/ml, 100 μl) by incubation at 4°C overnight and blocked with 5% BSA in TBS. Affinity-selected phage were added to the wells and allowed to bind at 37°C for 1 h. After the unbounded phage was removed, the bound phage was detected by incubation with peroxidase-labelled murine anti-M13 antibodies. The bound peroxidase was determined by incubation with O-phenylenediamine dihydrochloride and the reaction was determined in an ELISA reader at OD_492_. All the assays were carried out in triplicate and the error bars indicate standard deviation.

**Figure 5:**
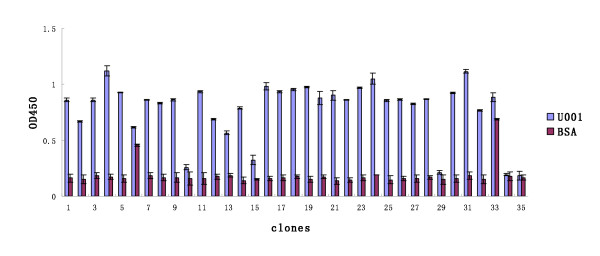
**Binding specificity of selected phage from the Ph.D.-C7C library**. Wells of 96-well microtitre plates were coated with mAb U001 and BSA (10 μg/ml, 100 μl) by incubation at 4°C overnight and blocked with 5% BSA in TBS. Affinity-selected phage were added to the wells and allowed to bind at 37°C for 1 h. After the unbounded phage was removed, the bound phage was detected by incubation with peroxidase-labelled murine anti-M13 antibodies. The bound peroxidase was determined by incubation with O-phenylenediamine dihydrochloride and the reaction was determined in an ELISA reader at OD_492_. All the assays were carried out in triplicate and the error bars indicate standard deviation.

To map the neutralising epitopes of urease B that were recognised by mAb U001, the Ph.D.-12 and Ph.D.-C7C libraries were screened with this purified mAb. After three rounds of biopanning, the ratio of output/input increased (Table 1) and 70 clones (35 each from the Ph.D.-12 and Ph.D.-C7C libraries) were randomly selected. When tested by ELISA for their immunoreactivity with mAb U001, 29 dodecapeptide clones and 28 heptapeptide clones gave positive signals (Figures 4 and 5) with BSA cross-reactivity, and sequences of 15 clones for each library were tested. The deduced amino acid sequences of the corresponding inserts were identified as six different sequences for the Ph.D.-12 library and five for the Ph.D.-C7C library (Table 2). The deduced amino acid sequences of selected phage clones were aligned and analysed. The amino acid sequences appearing in more than three different selected clones and matched with urease B protein were grouped as the consensus residues and +summarised with bold letters (Table 2). Although these clones did not match the urease B protein sequence, they shared extensive homology with each other and were clustered and classified as the conformational epitopes of urease B. Peptide EXXXHDM from the Ph.D.-12 library and EXXXHSM from the Ph.D.-C7C library showed a good match with the urease B protein at 347-353 aa. Amino acids E, D, H, M and S are key residues of the urease B epitope.

**Table 1:** Enrichment of phage displaying epitope by panning over mAb U001 from ph.D.-12 and Ph.D.-C7C

Library	Cycle	coating mAb (mg/L)	Washing (TBST)	Input phage (pfu)	Output phage (pfu)	Recovery rate (%)
	I	100	0.1%	1.5×10^11^	8.4×10^3^	5.6×10^-6^
Ph.D.-12	II	10	0.3%	1.5×10^11^	8.0×10^4^	5.3×10^-5^
	III	1	0.5%	1.5×10^11^	3.0×10^5^	2.0×10^-4^

	I	100	0.1%	1.5×10^11^	7.6×10^3^	5.4×10^-6^
Ph.D.-C7C	II	10	0.3%	1.5×10^11^	7.2×10^4^	4.8×10^-5^
	III	1	0.5%	1.5×10^11^	3.5×10^5^	2.3×10^-4^

**Table 2:** The sequences of ph.D.-12 and ph.D.-C7C phage-displaying peptides from the recombinant phage clones randomly selected through biopanning

Library	**Clone no**.	Sequence
	UreaseB protein	**E **D T L **H D M **G I F S I (347-358)
	D1~D3, D5,D6,D8,D9, D11,D13	**E **H W S **H D M **F S P G D
	D10,D15	**E **V S **L H D M **N L A T H
Ph.D.-12	D4	K W L G **H D M **I M S G T
	D7	F N **T **K **H D M **Q G D T S
	D12	**E **H N D F P **M **Y T W R P
	D14	T T T H F L A T K F Y K

	H1~H2, H4,H6~H9,H11,H13,H15	**E **K L K **H S M**
	H5,H10	T K T W Q **S M**
**Ph.D.-C7C**	H3	M S L L G H K
	H12	K H G L L S M
	H14	M S Q D G H T

**Figure 6:**
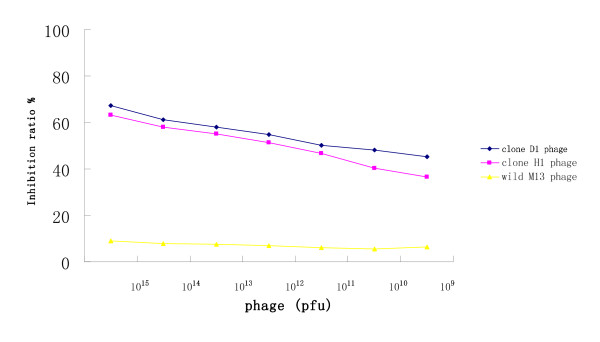
**Phage inhibition of mAb U001 binding to urease B protein**. Wells of microtitre plates were coated with urease B protein and incubated with the mAb U001 (0.5 μg) plus increasing amounts (10^9^-10^15 ^pfu) of the selected phage (clones D1 and H1). The binding was monitored by OD_492 _measurements after reaction with the peroxidase-conjugated goat anti-mouse IgG. The inhibition ratio was determined by the following equation: [(activity without Ab - activity with Ab)/(activity without Ab)] × 100.

Competitive ELISA was performed to confirm the specificity of the positive clones. The results showed that the recombinant phage D1 clone (with **E**HWS**HDM**FSPGD sequence) and phage H1 clone (with **E**KLK**HSM **sequence) all inhibited the mAb U001 binding to recombinant urease B protein, whereas wild-type M13 phage had no effect (Figure 6).

### Immune response induced by the selected phage clones

**Figure 7:**
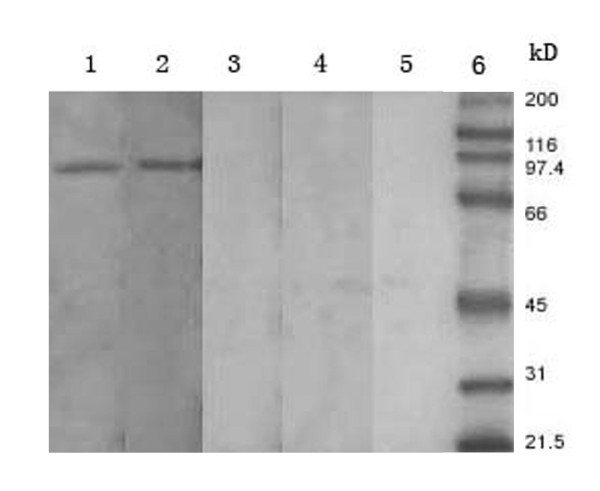
**Western blot analysis of antiserum induced by phagotopes**. Two phage (clones D1 and H1) were used to immunise BALB/c mice through intraperitoneal administration. For each immunisation, 10^12 ^pfu phage (in 50 μl) was used, with TBS and wild-type M13 phage as controls. Three mice were immunised with each sample and pre-immune sera were obtained. The mice were bled once (pre-booster), and they were given a booster in the same manner. The immune mice were bled every 7 days after booster immunisation, and western blotting was performed to test each serum sample. 1: serum from D1 phagotope; 2: serum from H1 phagotope; 3: pre-immune serum; 4: serum from wild M13phage; 5: serum from TBS.

**Figure 8:**
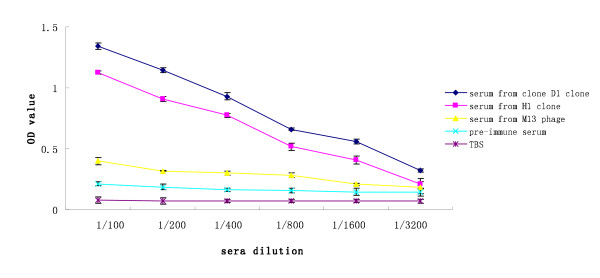
**Serum antibodies induced by phagotopes**. Two purified phage clones (D1 and H1) were used to immunise BALB/c mice through intraperitoneal administration. For each immunisation, phage (10^12 ^pfu/50 μl) were used, with TBS and wild-type M13 phage as controls. Three mice were immunised with each sample and pre-immune sera were obtained. The mice were bled once (pre-booster), and they were given a booster in the same manner. The immune mice were bled every 7 days after the booster immunisation, and triplicate ELISAs were performed to test each serum sample with urease B as coated protein. All assays were carried out in triplicate and the error bars indicate standard deviation.

To evaluate the immune responses induced by the selected mimotopes as vaccine candidates, phage clones D1 and H1 were chosen to immunise BALB/c mice by intraperitoneal injection. Western blot analysis confirmed urease B recognition by the antisera (raised by phage clones and the controls; Figure 7). The sera from clone D1 (lane 1) and clone H1 (lane 2) reacted specifically with urease B protein at ~92 kDa, whereas the pre-immune serum (lane 3), and the serum induced by wild-type M13 phage (lane 4) or TBS (lane 5) were negative. Triplicate ELISA measurements demonstrated significant serum responses against urease B for each sample and specific antibody responses from clone D1 and H1 were higher than others. The immune response to wild-type M13 phage control was very low (Figure 8).

### Alignment results from MIMOX

**Figure 9:**
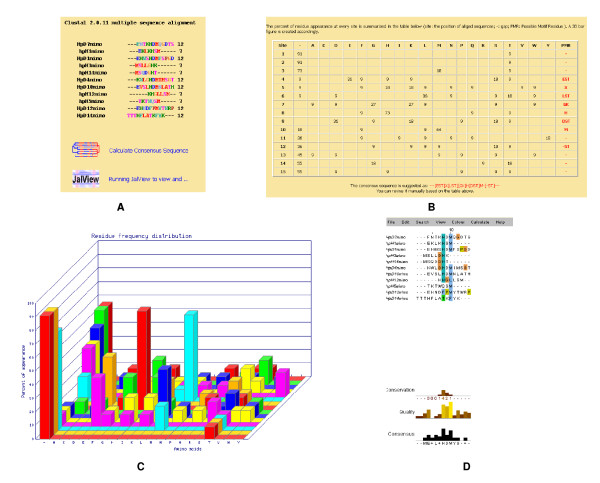
**Mimotopes selected out with U001 were input and aligned with ClustalW**. (A) Alignment of multiple sequences by ClustalW. (B) The frequency of a given amino acid at each position of the alignment was calculated and displayed in a table. (C) A 3D bar figure was created. The x axis represents the 20 amino acid types and gap, whereas the y axis is the frequency of occurrence and the z axis is the position of the aligned sequences. (D) The alignment could be managed with JalView.

Computer-based analysis was performed to define the consensus amino acid residues of the mimotopes. Mimox wrapped ClustalW to align a set of mimotope sequences that were analysed using an embedded version of JalView. Figure 9 shows the resultant analysis, which identified E, H, D and M as the consensus amino acid residues in the aligned mimotopes.

## Discussion

Investigators have demonstrated previously that peptide mimotopes obtained from phage epitope libraries or by chemical synthesis can bind antibodies raised against native structures. In fact, peptide mimotopes have been found for most, if not all, anti-carbohydrate mAbs [16]. Moreover, peptide epitopes can mimic glycosphingolipids and oligonucleotide structures [15,25]. In the case of mimotope immunisation, several studies have shown effective responses *in vivo *[26,27]. Furthermore, protective immune responses by mimotope immunisation have been verified in many infectious diseases [7,28-30].

In the field of vaccine design, the phage-displayed mimotopes have recently been shown to be possible vaccine components that do not necessarily represent the structural equivalents of the original antigen, but provide functional images that could replace the original epitopes for vaccine development [31-33]. Application of phage display technology to elucidate the essential chemical features of conformational or discontinuous epitopes that are recognised by some disease-related antibodies can provide important information about the molecular mechanisms of these diseases. The selected sets of peptides might also lead to the future design of more effective reagents for therapeutic and/or diagnostic purposes. The main advantage of the phage-based approach is that it does not produce a consensus sequence of a large pool of peptides that share a common property, but rather yields a list of peptide sequences, each of which is endowed with the same property. Alignment and comparison of the amino acid sequences of the selected clones can then determine one or more consensus sequences. This approach enabled us to obtain the epitope profile of *H. pylori *in an efficient manner. The profiles not only reveal the important roles of each antigen, but also provide the epitope structures within the antigenic protein that is involved in the induction of successful immune responses.

In this study, we first cloned and expressed urease B of *H. pylori*, which was then used as an immunogen to produce mAbs against this recombinant antigen by hybridoma technology. We obtained two types of mAb against different epitopes of urease B and observed that mAb U001 strongly inhibited its enzymatic activity, whereas mAb U002 did not induce this inhibitory activity. Therefore, mAb U001 could be beneficial for the prevention of bacterial growth and attachment to the gastric mucosa, thus, we identified the neutralising epitope of this mAb via two phage display libraries (Ph.D.-12 and Ph.D.-C7C). The two mimotopes of urease B were characterised via affinity screening, binding, competitive inhibition and DNA sequencing. Most clones from the Ph.D.-12 library share the core EXXXHDM sequence, therefore, we consider that EXXXHDM might be a motif of urease B because clone D1 (with this sequence) competitively inhibited U001 binding to urease B and induced an immune response in mice. Clone H1 (EKLKHSM sequence) from the Ph.D.-C7C library appeared at a high frequency and had the same function as clone D1. The mimotopes EXXXHDM and EXXXHSM, which are highly homologous with urease B residues 347-353 aa are also adjacent to a neutralising epitope on urease B (321-339 aa), as reported previously [34]. Li *et al. *have identified a B-cell-neutralising epitope of urease B (211-225 aa) [35]. These observations suggest that there are predominant antigenic epitopes on the urease B protein that are effectively targeted.

The mimotopes selected from the above-mentioned phage display libraries are not completely homologous with the known amino acid sequence of urease B. Many researchers have demonstrated similar results to ours using antibodies as a tool to screen phage display library mimotopes for other target protein antigens. One reason for the disparity in mimotope urease B sequences might be that the epitopes that are recognised by the generated mAbs could be a linear or conformational epitope that consists of a discontinuous amino acid sequence. In the latter case, the discontinuous amino acids could be in close spatial proximity due to the folding of peptides. We therefore speculate that amino acids E, D, H, M and S are key residues for the urease B epitope. Our results suggest that sequences that contain EXXXHDM and EXXXHSM with potential antigenicity and immunogenicity should be further studied for their use in the development of a diagnostic reagent, and vaccine that targets *H. pylori *urease B. It is particularly interesting that H3 and H12 screened from the Ph.D.-C7C library (Table 2) are exactly the same but in inverse order. Unlike the Ph.D.-12 phage display library, the randomised sequence of the Ph.D-C7C library is flanked by a pair of cysteine residues. Under non-reducing conditions, the cysteine residues spontaneously form a disulfide cross-link, which results in phage display of cyclized peptides. Disulphide-constrained peptide libraries have previously proven useful in identification of structural epitopes. From this study, we presume that the epitope recognised by mAb U001 is a structural epitope. Amio acid S and M, which are also included in H1, are the key amino acids that constitute this structural epitope.

Although the immune systems in mice and humans differ, it is still informative to check the immune responses and evaluate the potential of these experimental vaccine candidates in mice. Recombinant phage can display epitopes and also bear significant adjuvant activity [36], therefore, we directly used phagotopes as antigens to immunise mice. Our findings showed that the phage-displayed epitope was able to mimic immunological properties of the native epitope on *H. pylori *urease B. Furthermore, using phage-displayed epitopes as antigens, we were able to detect the antibody in the sera from immunised animals. The results suggest that the phagotopes also provide a convenient and economic approach to prepare antigens, and could be applied in an ELISA-based method for the rapid diagnosis of *H. pylori *infection.

## Conclusions

We demonstrated the feasibility of identifying important mimotopes of urease B for vaccine development by screening phage-displayed random peptide libraries with mAb. The results also indicate the potential application of phagotopes as alternative vaccine components. Comparison of mimotopes selected from a peptide library with mAb and the original antigen sequence could lead to a better understanding of the molecular mechanisms that participate in the immune response and to the design of specific peptides for diagnostic purposes and vaccine development.

## Methods

### Bacterial strains and growth conditions

*H. pylori *strain NCTC11639 was obtained from the Department of Microbiology, Southern Medical University (Guangzhou, China). This cell line was routinely cultured on Columbia agar plates supplemented with 7% saponin-lysed horse blood, 0.004% triphenyltertrazolium chloride, and Dent selective supplement (Oxoid, Basingstoke, UK) for 3 days at 37°C under a micoaerophilic atmosphere (5% O_2_,15% CO_2 _and 80% N_2_). A colony was picked up and inoculated into 20 ml Brucella broth (Becton Dickinson, Cockeysville, MD, USA) that contained 0.1% β-cyclodextrin supplemented with 5% (v/v) horse serum for 24 h. Two millilitres of culture medium was transferred to 40 ml fresh medium and the same processes were repeated twice. One millilitre of the incubated cell-containing medium (mostly spiral rather than coccoid bacteria) was plated on Brucella agar (Becton Dickinson) that contained 7% (v/v) defibrinated horse blood, and cultured for an additional 3 days at 37°C in a microaerophilic atmosphere. Bacterial cells were harvested and washed twice with cold PBS (25 mM sodium phosphate, pH 7.2, 0.9% NaCl, pH 7.0). The cells were sedimented by centrifugation (5,000×*g *for 10 min at 4°C) and stored at -80°C.

### Cloning, expression and enzymatic activity of recombinant urease B protein

Genomic *H. pylori *DNA was prepared using a DNA extraction kit (Takara, Belmont, Japan) according to the manufacturer's instructions. Extracted genomic DNA was then used as the template for amplification of the urease B coding region using the Taq DNA polymerase PCR kit (Takara). Two primer sequences that corresponded to the 5' and 3' end of coding gene plus *Eco*RI and *Sal*I restriction sites were P1: 5'-TGCGAATTCAAAAAGATTAGCAGAAAA-3' and P2: 5'-GGGTCGACGAAAATGCTAAAGAGTTG-3'. The PCR products were cloned into an *Eco*RI- and *Sal*I-digested pGEX-4T-1 expression vector (Amersham Biosciences). The plasmid was then transformed into *E. coli *strain TOP10 (Invitrogen BV, Leek, The Netherlands) and urease B expression was induced with 1 mM IPTG when the cells were in log phase growth at 37°C. After 4 h, the cells were harvested by centrifugation at 7000 rpm/min for 10 min and washed with ice-cold PBS that contained 5 mM EDTA and 2 mM PMSF. All subsequent procedures were performed at 4°C.

To purify recombinant urease B, the cells were resuspended in PBS that contained 5 mM EDTA and 2 mM PMSF and lysed by sonication on ice, and centrifuged at 10,000 rpm for 20 min to sediment cell debris. The supernatant was applied to Glutathione Sepharose 4B (Pharmacia, Uppsala, Sweden) to purify urease B protein according to the manufacturer's protocol. Urease B samples were analysed by electrophoresis on a 10% SDS-polyacrylamide gel. The gels were stained for protein with 0.25% Coomassie Blue or transferred to a nitrocellulose membrane (Millipore, Bedford, MA, USA) and probed with diluted human sera (1:50 dilution with PBS) from a patient diagnosed with *H. pylori *infection (positive serum) and a normal donor without infection (negative serum). Detection was performed with a goat anti-human antibody linked to horseradish peroxidase (1:10,000) followed by staining with 3-amino-9-ethylcarbozole (Sigma, St Louis, MO, USA) as a chromogen.

### Production of the mAb and urease B inhibition test

BALB/c female mice, 4-6 weeks old, were purchased from the Animal Center of Southern Medical University. The mice were immunised by intraperitoneal injection with 50 μg purified urease B-GST, which was emulsified with an equal volume of Freund's complete adjuvant. Three additional booster injections were administered using incomplete adjuvant every 2 weeks. Three days after the last injection, the spleen cells were harvested and fused with the Sp 2/0 mouse myeloma cell line. After 10-14 days, the culture supernatants were screened for positive mAbs against recombinant urease B-GST by ELISA. In the screening process, the mAbs that reacted with urease B-GST but not with GST were selected. Positive colonies were subcloned twice by limiting dilution. Ascitic fluid was obtained from mice primed with a 0.5-ml intraperitoneal injection of Pristane and then injected with 10^6 ^hybridoma cells. The class and subclass of each mAb were determined with a mouse monoclonal antibody isotyping kit (HyCult Biotechnology, Uden, The Netherlands). The mAbs were purified using protein G-Sepharose 4FF (Pharmacia) according to the manufacturer's instructions, to remove components that might have interfered with the biopanning experiments.

The antibody titres were determined by ELISA. Purified urease B-GST antigen was coated on the microtitre plates (100 μl/well, 5 μg/ml in coating solution, 0.1 M sodium bicarbonate, pH 9.6) (Corning, Corning, NY, USA) and incubated at 4°C overnight. The supernatants of the cell culture as well as the ascitic fluids were incubated with pre-coated urease B-GST (100 μl/well) for 2 h at 37°C. Goat anti-mouse IgG HRP (1:10,000 dilution with washing buffer) was used to detect urease B-GST mAbs and O-phenylenediamine dihydrochloride (Pierce Chemicals) was used as substrate for HRP and the reaction was monitored at an absorption of 492 nm using a microplate reader (Model 550; Bio-Rad).

The affinity constant (K_a_) of mAbs was determined by using a method that was developed by Beatty [37]. Urease B-GST protein was coated on the ELISA plates at three different concentrations ([Ag], [Ag'], and [Ag'']) followed by incubation with each mAb at different concentrations. The K_a _of each mAb was calculated based upon the sigmoid curves that represented the relationship between the concentration of mAb and the OD values. The antibody concentration at half maximal absorption for each curve was obtained by extrapolating the curves and named ([Ab], [Ab'], and [Ab'']). The Ka was calculated using the following equation: K = (n-1)/2(n [Ab'] -[Ab]), where n = Ag/Ag'.

Using competitive ELISA, the topology of the epitopes that were recognised by the mAbs was studied. Competitive tests compared the ability of the various unlabeled mAbs (competitors) to compete with the peroxidase-conjugated mAbs for binding to urease B that was immobilised on ELISA plates, in which saturating amounts of unlabelled mAbs were added to the plates coated with urease B protein before addition of labelled mAbs. Different concentrations of each competitor mAb in PBS, which contained a constant concentration of a labelled mAb, were co-incubated with immobilised urease B for 40 min at 37°C. Following extensive washing with PBS, the plates were developed with O-phenylenediamine dihydrochloride (Pierce Chemicals) as mentioned above. Percentage inhibition was finally estimated based on the following calculation: percentage inhibition = [(OD without competitor) - (OD with competitor)/OD without competitor] ×100.

Purified *H. pylori *urease B was prepared. Stored cell pellets were thawed, resuspended in 10% n-octyl β-D-glucopyranoside (Wako Pure Chemical Industries), vortexed five times for 10 s each, and centrifuged at 10,000 × *g *for 20 min at 4°C). DL-Dithiothreitol (DTT; 0.1 mM) (Sigma) was added to the supernatant, and the mixture was applied to a PD-10 column (Sephadex G-25; Pharmacia Biotech, Uppsala, Sweden), and the eluate was dialysed against PBS that contained DTT. The eluate was applied to a column (Shoei Works Co.) with Affi-Gel Hz hydrazide gel (Bio-Rad) coupled to D12(S3) overnight in a cold room. The column was washed with PBS that contained DTT, and elution was performed with 5 M urea in 0.1 M potassium phosphate buffer (pH 6.5) that contained 0.1 mM DTT. The eluate was dialysed with the PD-10 column. The amount of protein was determined by Lowry's method.

The *H. pylori *urease inhibition test was carried out using the mAbs prepared above. Urease (25 μl) was incubated with 25 μl anti-urease B epitope mAb U001, U002 (equivalent to 0-25 μg) in 96-well microtitre plates overnight at 4°C, with PBS as the control. On the following day, 50 μl 50 mM phosphate buffer (pH 6.8) that contained 500 mM urea, 0.02% phenol red, and 0.1 mM DTT was added to each well. The colour development was monitored at 550 nm with a microplate reader (Model 550; Bio-Rad). Percentage inhibition was determined by the following equation: [(activity without Ab - activity with Ab)/(activity without Ab)] × 100.

### Phage display library screening using specific mAb as a target molecule

The Ph.D.-12 and Ph.D.-C7C libraries were obtained from New England BioLabs. They were combinatorial peptides that were fused to the minor coat protein (pIII) of M13 phage, and consisted of about 1.5×10^13 ^and 2×10^13 ^pfu/ml electroporated sequences, respectively. Three rounds of biopanning were undertaken according to the manufacturer's instructions, with some modifications. A plate well was coated with 100 μg/well of anti-urease B mAb U001 in 0.1 M NaHCO_3_, pH 8.6, and incubated overnight at 4°C. The well was washed 10 times with TBST (50 mM Tris, 150 mM NaCl, pH 7.5, and 0.1% Tween-20), filled with 400 μl blocking buffer (0.1 M NaHCO_3_, pH 8.6, 5 mg/ml BSA, and 0.02% NaN_3_), and incubated for 1 h at 4°C. In the first round of biopanning, 10 μl phage (1.5×10^11^) from the initial library, in 100 μl TBST, was incubated with the well-bound mAb for 1 h at room temperature, under rocking conditions. After repeated washes with TBST, the bound phage was eluted from the well with 100 μl elution buffer (0.2 M glycine-HCl, pH 2.2). After neutralisation with 15 μl 1 M Tris-HCl, pH 9.1, the eluted phage were amplified by infecting 20 ml of a 1:100 dilution of an overnight culture of *E. coli *ER2738. The culture was incubated for 4.5 h at 37°C with vigorous shaking. The supernatants were obtained and precipitated with PEG/NaCl.

In the second and third rounds of biopanning, 20% of the amplified phage from the preceding round was incubated with mAb U001 overnight at 4°C as described above. The procedures were identical to the first round except that the coated mAb concentration was 10 mg/l and 1 mg/l, and the washing steps were carried out with 0.3% and 0.5% TBST for the second and third round of biopanning, respectively.

### Binding specificity of the selected phage by ELISA and DNA sequencing

Ninety-six-well plates were coated with mAb U001 and BSA (10 μg/ml, 100 μl) by incubation at 4°C overnight, and blocked with 5% BSA in TBS. Affinity-selected phage were added to the wells and allowed to bind at 37°C for 1 h. After the unbounded phage was removed with 5% TBST, the bound phage were detected by incubation with peroxidase-labelled murine anti-M13 antibodies (Promega, Madison, WI, USA). The bound peroxidase was determined by incubation with O-phenylenediamine dihydrochloride (Pierce Chemicals) in buffer (30 mM citrate, 70 mM Na_2_HPO_4_, and 0.02% H_2_O_2_, pH 5.5). After the reaction was stopped by the addition of 3 N HCl, A_492 _was determined with an ELISA reader (model 550; Bio Rad). All the assays were carried out in triplicate.

The phage from the third biopanning eluate were cloned and amplified for DNA sequencing and immunoanalysis. The nucleotide sequence of the gene III insert was determined as per the instruction manual. The amino acid sequence of the insert was deduced from the nucleotide sequence and was compared with native urease B sequence using DNAMAN version 6.0 software. The sequences that appeared >3 times among the selected phage clones were classified as the consensus sequence. The aligned amino acid sequences shared by three (or more) identical amino acids within the dodecapeptides (heptapeptides) were determined as the mimotopes of the matched protein sequences.

Competitive ELISA analysed the binding specificities of the recombinant phage to the screened mAb U001. The wells of microtitre plates were coated with urease B protein and incubated with mAb U001 (0.5 μg) plus increasing amounts of the selected phage. The binding was monitored by OD_492 _measurements after reaction with the peroxidase-conjugated goat anti-mouse IgG (Sigma).

### Animal immunisation and preparation of antisera

To evaluate the potential of the selected mimotopes as experimental vaccine candidates, two purified phage clones (D1 and H1) were used to immunise female inbred specific-pathogen-free BALB/c mice through intraperitoneal administration. For each immunisation, phage (~10^12 ^pfu/50 μl) was used, and TBS and wild-type M13 phage were used as the negative controls. Three mice were immunised with each sample and pre-immune sera (negative control) were obtained. The mice were bled once (pre-booster), and then they were given booster injections in the same manner. The immune mice were bled every 7 days after the booster immunisation, and ELISA and western blotting were performed to test each serum sample for *H. pylori *urease B-specific antibody activity.

### Analyzing mimotopes with MIMOX

MIMOX (http://immunet.cn/mimox/intro.html#7) is a web tool for bioinformatics analysis of peptide sequences derived from phage display. The interface ClustalW can align a set of mimotope sequences and deduce consensus sequences based on the aligned mimotopes through a simple statistical method. A set of mimotopes obtained above (including 6 dodecapeptides and 5 heptapeptides) was input and aligned with all parameters as defaults. The alignment can be viewed and managed through JAIView.

## Authors' contributions

YL performed the majority of the bench work except for the preparation of mouse antibodies against urease B. YW also contributed the majority of the work except for screening the phage library. DP participated in cloning and expressing recombinant urease B. YJ and LZ performed ELISA and western blotting. ML and JL performed the culture of *H. pylori *and immunised the animals. YN and ML designed the experiments. YL wrote the manuscript and all authors have read and approved the final manuscript.
